# Fatalism, Social Support and Self-Management Perceptions among Rural African Americans Living with Diabetes and Pre-Diabetes

**DOI:** 10.3390/nursrep11020024

**Published:** 2021-04-12

**Authors:** Laurie Abbott, Elizabeth Slate, Lucinda Graven, Jennifer Lemacks, Joan Grant

**Affiliations:** 1 College of Nursing, Florida State University, Tallahassee, FL 32306, USA; lgraven@fsu.edu; 2 Department of Statistics, Florida State University, Tallahassee, FL 32306, USA; eslate@fsu.edu; 3 School of Kinesiology and Nutrition, University of Southern Mississippi, Hattiesburg, MS 39406, USA; jennifer.lemacks@usm.edu; 4 School of Nursing, University of Alabama at Birmingham, Birmingham, AL 35294, USA; grantj@uab.edu

**Keywords:** rural, diabetes, health promotion, fatalism, risk reduction, fatalism, social support, self-management

## Abstract

Diabetes is a public health problem and a major risk factor for cardiovascular disease, the leading cause of death in the United States. Diabetes is prevalent among underserved rural populations. The purposes of this study were to perform secondary analyses of existing clinical trial data to determine whether a diabetes health promotion and disease risk reduction intervention had an effect on diabetes fatalism, social support, and perceived diabetes self-management and to provide precise estimates of the mean levels of these variables in an understudied population. Data were collected during a cluster randomized trial implemented among African American participants (*n* = 146) in a rural, southern area and analyzed using a linear mixed model. The results indicated that the intervention had no significant effect on perceived diabetes management (*p* = 0.8), diabetes fatalism (*p* = 0.3), or social support (*p* = 0.4). However, the estimates showed that, in the population, diabetes fatalism levels were moderate (95% CI = (27.6, 31.3)), and levels of social support (CI = (4.0, 4.4)) and perceived diabetes self-management (CI = (27.7, 29.3)) were high. These findings suggest that diabetes fatalism, social support, and self-management perceptions influence diabetes self-care and rural health outcomes and should be addressed in diabetes interventions.

## 1. Introduction

Cardiovascular disease (CVD) affects 48% (121.5 million) of all people living in the United States and is the leading cause of death [1,2]. African American (AA) and rural southern populations bear a disproportionately higher burden of CVD and stroke compared with other groups [2,3,4,5,6,7,8,9,10,11]. For example, AA men (60.1%) and women (57.1%) have a greater prevalence of CVD than White men (50.6%) and women (43.4%) [1,2,3,4,5,6,7,8,9,10,11]. As a major contributory risk factor, diabetes influences CVD development and progression and has been associated with poor health outcomes, increased mortality, and health disparities [2,12,13,14]. Relatedly, AA men and women are more likely to be diagnosed with diabetes and pre-diabetes than other groups [15].

Uncontrolled diabetes is a significant CVD risk factor among rural populations that has been associated with inadequate disease management behaviors, poor dietary patterns, ineffective medication adherence, and limited physical activity [16,17,18]. Diabetes and other chronic diseases are especially prevalent among populations living in rural areas of the southeastern United States, especially rural, southern AAs who have poorer health and the lowest reported life expectancy rates in the nation [4,5,6,7,9]. Such rural health disparities have been associated with resource limitations, disease knowledge deficits, and fewer primary and preventive health care services [2,4,6,9,10,19,20].

Individual psychosocial factors such as diabetes fatalism, social support, and perceived diabetes self-management also have a role by influencing diabetes self-care behaviors and outcomes [21,22,23]. As a subscale of diabetes fatalism, diabetes distress is described as the emotional frustration resulting from daily living with diabetes and the lifestyle disruptions from managing the condition [24]. Elevated diabetes distress has been associated with increased fatalism, decreased self-care, and lower glycemic control, as evidenced by higher HbA1C levels among people managing diabetes [21,24], including rural populations [17]. In comparison, having less psychological distress related to diabetes fatalism was associated with greater self-efficacy in managing diabetes and improved diabetes self-care [25]. Diabetes fatalism has been defined as a psychological pattern involving components of distress, religious and spiritual coping, and perceived self-efficacy [24]. Social support has a positive influence on diabetes self-management, weight control, and health outcomes [26,27,28,29], and it enhances resilience [30] and plays a role in diabetes prevention [31,32]. Self-efficacy for managing diabetes can either be positively or negatively related to diabetes self-care activities such as diet, exercise, and taking medications [33].

More information regarding these psychosocial determinants of diabetes outcomes is needed to improve diabetes self-care and management among diverse populations [17,34,35]. Interventions intended to promote healthy lifestyles have shown efficacy in reducing disease risk among AA populations [14]. However, research is lacking about psychosocial factors of glycemic control such as diabetes fatalism, social support, and perceptions of diabetes self-management among African Americans living in the rural southern states, where diabetes and CVD disparities are greatest [36]. The purposes of this study were to perform secondary analyses of existing clinical trial data to determine whether a diabetes health promotion and disease risk reduction intervention had an effect on diabetes fatalism, social support, and perceived diabetes self-management and to provide precise estimates of the mean levels of these variables in this rural population.

The theoretical framework that guided this study was the Information–Motivation–Behavioral Skills Diabetes Self-Care (IMB-DSC) model [37]. The model postulates that diabetes self-care behaviors and, ultimately, glycemic control are directly influenced by factors including diabetes knowledge, diabetes fatalism, and social support [37]. The self-care behaviors described by the model include general and specific diet, foot care, and blood sugar testing. Having more diabetes knowledge, less fatalism, and greater social support have been associated with increased likelihood of performing the diabetes self-care behaviors directly linked to glycemic control [37].

## 2. Materials and Methods

### 2.1. Study Design

This study is a secondary analysis of data collected during a cluster randomized trial to test the culturally relevant “Project POWER” program in a rural, southern AA population [38]. This study was approved by the Institutional Review Board (IRB) at Florida State University before any study activities were conducted. Outcomes by sociodemographic characteristics including gender, age, diabetes or pre-diabetes diagnosis, and employment status were also explored.

### 2.2. Participants and Sample Size

Participants were recruited through regional churches, and because individuals within the same church may share characteristics that could induce correlation among their outcomes [39], churches, rather than individuals, were randomized to either the intervention or control group. The minimum sample size was calculated using a conservative intra-cluster correlation value (*r* = 0.008) that had been reported in previous health education research [40] and was determined to detect a medium standardized effect size (*d* = 0.50) [41]. For sufficient power (80%), at least 71 participants, including 10% for attrition, and five participating churches was needed for each of the two study groups.

The first step in the recruitment plan involved engaging rural church pastors by telephone and discussing the study purpose and research activities. Pastors interested in having their congregations participate gave permission for this study to be conducted on church grounds. The participating churches were randomized using a five-digit random number sequence to allocate assignment to either the intervention (even number) or control (odd number) group. The starting number in the sequence was randomly selected, and each number group was checked to ensure the parity imbalance was no greater than two. The pastors were informed of the church group allocation, posted flyers, and made announcements within their congregations about this study and provided the first session date. The pastor discussed the inclusion criteria with congregants, which included self-identification as African American, at least 22 years of age, previously diagnosed with diabetes or pre-diabetes, and the ability to understand and speak English. During the first session, the study purpose, activities, and inclusion requirements were discussed by research staff, and those eligible and willing to participate provided written informed consent.

### 2.3. Measures

Data were collected from each participant in both the intervention and control groups at baseline and three weeks later. The measures included a sociodemographic survey that was used to collect data regarding age, gender, employment, education, diabetes or pre-diabetes diagnosis, history of heart or kidney disease, diabetes management regimen, family history of diabetes or heart disease, and diagnosed diabetic retinopathy.

The IMB-DSC framework [37] guided the choice of measures that were used to collect data in the parent study and analyzed in this secondary analysis. The instruments gathered self-reported information about diabetes fatalism, social support, and perceived diabetes self-management. The Diabetes Fatalism Scale [24] is a 12-item, 6-point Likert scale that measures three constructs of diabetes fatalism: emotional distress, religious and spiritual coping, and perceived self-efficacy. Scores range from 12 to 72, with higher scores representing greater diabetes fatalism. The internal consistency (α = 0.80) for the overall measure was adequate as were the subscale scores for emotional distress (α = 0.86), religious and spiritual coping (α = 0.77), and perceived self-efficacy (α = 0.77). The 20-item Medical Outcomes Study Social Support Survey [42] was used to measure social support. The instrument has one fill-in-the-blank item for number of close friends and relatives and 19 Likert scale, 5-point options between “None of the Time” (1) to “All of the Time” (5). In addition to, the total score (range, 19–95), the measure yields four social support subscales (Tangible Support, Emotional/ Informational Support, Affectionate Support, and Positive Social Interaction) which had excellent (α = 0.91–0.97) internal consistency. The Perceived Diabetes Self-Management Scale (PDSMS) [33] measures perceptions of diabetes self-management and is an 8-item, Likert scale-type instrument with responses that range from “Strongly Disagree” (1) to “Strongly Agree” (5). The measure allowed a range of total possible scores from 8 to 40 and had adequate internal consistency (α = 0.83) in previous research. Higher scores were associated with greater confidence in self-managing diabetes-related goals and activities.

### 2.4. Intervention Description

The intervention group participants in churches received the “Project POWER” curriculum developed by the American Diabetes Association as a culturally relevant diabetes health promotion program for African American adults in church settings. The intervention was delivered over three sessions by the same advanced public health nurse on days decided by the church pastors. The content was similar to other diabetes risk reduction programs. During the first session, information was presented about diabetes types, risk factors, and symptoms of hyperglycemia and hypoglycemia. The second session discussed managing glycemic control through diet and exercise, and the third session included strategies to manage diabetes and reduce risk for CVD, kidney disease, and stroke. The strategies to encourage participant engagement and enhance learning involved interactive discussion, handouts, games, and roleplays. Those in the waitlist control group received a diabetes self-care brochure developed and published by the American Diabetes Association.

### 2.5. Data Analysis

Participant sociodemographic characteristics are summarized for each group by mean and standard deviations (for age) and proportions for the remaining categorical variables. The effect of the Project POWER intervention was assessed using a repeated-measures linear mixed model (LMM) incorporating a random effect for church, thus accommodating within-cluster correlation in addition to the within-subject pre-post dependency. This model included fixed effects for time, group, and the group by time interaction, the last of which quantified the Project POWER intervention effect. We report the point estimates and 95% confidence intervals for the change from pretest to posttest for each study group and the effect of the time-by-group interaction. Because the data do not support an intervention effect, we additionally refit these mixed models retaining only the main effect for time in order to pool study groups and obtain precise estimates of the mean outcomes (diabetes fatalism, social support and perceived diabetes self-management) adjusted for time and appropriately incorporating the dependence structure. Since the IMB-DSC model indicates that social support and diabetes fatalism are important determinants of diabetes self-care, the precise estimates provide information about these study variables in the participating rural sample. Further analyses assessed differences by gender, age, diabetes or pre-diabetes diagnosis, and employment status by the significance of the F test for these main effects when added to the model. All analyses used the intention-to-treat data set, which included all participants according to the randomized assignment of their church, and were performed using SAS version 9.4. (SAS, Cary, NC, USA) Confidence intervals and *p*-values are reported without adjustment for multiplicity.

## 3. Results

The data collected from 146 participants (randomized as 71 to control and 75 to Project POWER) during a cluster randomized intervention study were analyzed. There were no significant differences in baseline sociodemographic characteristics between the intervention and control groups except for employment status and history of diabetic retinopathy [38]. The ages of participants ranged from 26 to 91 years of age. As shown in Table 1, there were lower percentages of full-time employed participants in the intervention group (20.0%) compared with the control group (39.4%) and higher percentages of participants in the intervention group who were unemployed (72.0%) than those in the control group (49.3%). More people in the intervention group (*n* = 14, 18.7%) had been diagnosed with diabetic retinopathy than those in the control group (*n* = 2, 2.8%). There were also notable gender differences between groups. Fewer men were in the intervention group (*n* = 14, 18.7%) than the control group (*n* = 22, 31%), and there were more women in the intervention group (*n* = 61, 81.3%) than control (*n* = 49, 69%). There were lower percentages in the intervention group than the control group with a family history of diabetes (80.0% vs. 90.1%) and heart disease (80.0% vs. 90.0%). All subjects provided baseline assessments; two subjects in the control group and seven subjects in the intervention group did not provide the follow-up data.

The effect of assignment to the Project POWER intervention was not statistically significant for diabetes fatalism, social support, and perceived diabetes self-management, nor their subscales (Table 2). Although the intervention did not induce statistically significant between-group differences for these factors, the results showed slightly improved changes from pretest to posttest for the intervention group compared with the control group for most of the variables and their subscales. The total score for diabetes fatalism had a greater decrease for the intervention group (−3.15) than the control group (−1.34) indicating that the intervention may have had a slight impact in reducing fatalism, especially for the emotional distress (*p* = 0.06) subscale (Table 2). The total and subscale scores for social support and the total scores for the perceived diabetes self-management slightly improved though the changes were not statistically significant.

Overall, there were improved scores from baseline to follow up in perceived diabetes self-management and each of the two dimensions, including the subscales, though not all were statistically significant (Table 3). The estimated population mean diabetes fatalism total score, social support total score and perceived diabetes self-management score were 29.48 [95% CI = (27.63, 31.3)], 4.18 (3.99, 4.38) and 28.49 (27.68, 29.31), respectively. Estimates of the subscale population means are also given in Table 3. Select subscale means differed by gender, age, diabetes diagnosis, or employment status. Specifically, for diabetes fatalism, the emotional distress subscale varied by diagnosis status (diabetes diagnosis < pre-diabetes diagnosis, *p* = 0.034) and perceived self-efficacy varied by employment (Not employed < full time < part time, *p* = 0.05) and age (*p* = 0.04). For social support, the total score and both the emotional/informational and affectionate subscales varied by gender (males < females, *p* = 0.03, 0.02, 0.01, respectively).

## 4. Discussion

Uncontrolled diabetes is a modifiable risk factor for CVD and other chronic diseases that impacts health outcomes and contributes to rural disparities. Factors that affect diabetes self-care behaviors such as diabetes fatalism, and social support should be addressed in standard diabetes care [21,34]. For example, strategies to reduce diabetes-related distress among rural populations have shown efficacy in improving glycemic control, diabetes self-efficacy, and self-care behaviors [17]. Similarly, as depicted in the IMB-DSC model, reduced diabetes fatalism is associated with better diabetes self-care activities that are directly linked to glycemic control [37]. AAs living in rural, southern states are historically underserved and hard to reach in health promotion and other research efforts. The findings from this secondary analysis contribute information that can potentially facilitate the development of future culturally relevant diabetes interventions and influence program implementation considerations.

Overall, the time-adjusted means indicated that participants had moderate levels of diabetes fatalism (M = 29.48), high levels of social support (M = 4.18), and somewhat high levels of perceived diabetes self-management (M = 28.49) (Table 3). For diabetes fatalism, previous research [24] found that fatalism levels (M = 34.0, SD = 9.5) were relatively higher among a predominantly urban AA study group (64.9%) [21] than the levels reported for the rural AA participants of this study. It is important to note that social support serves as a buffer of the effects of psychological distress experienced by AAs [43]. Church-based relationships are especially important among AA communities because they provide beneficial social support resources [44], especially when received from family members and friends [45], promote help-seeking for diabetes care [28] and facilitate healthy behaviors including increased produce intake and physical activity [43,46]. However, social support may differ by geographic location. One study showed that rural AA women had less contact with family and fewer social engagement opportunities in church settings than their urban counterparts [47]. However, social capital may be more influential and resourceful among some rural communities compared with others. The findings of this study indicated that the participants had high levels of social support and that support could be relied upon at least “most of the time”. These differences suggest that more research is needed to understand social support in rural populations,

For perceived diabetes self-management, a previous study [43] among urban AA participants (56%) indicated a moderately high (M = 24.8, SD = 6.35) level of perceived diabetes self-management. Comparatively, the overall perceptions of diabetes self-management were higher among the rural, southern AA participants of the current study which suggested greater confidence in self-managing diabetes at home. The scores for both groups were moderately high and showed a slightly higher effect (b = 0.24) for the intervention group compared with the control group. This suggests that the intervention may have had some effect even though it was not statistically apparent. A possible reason that there were not greater between-group differences could be that the curriculum mainly concentrated on lifestyle factors such as diet, exercise, and medication adherence. It would be interesting, however, to compare whether the perceptions of self-care were congruent with the current diabetes self-management guidelines recommended by health professionals.

There were differences when exploring the sociodemographic characteristics of the study population. For example, the larger number of people in the intervention group diagnosed with diabetic retinopathy could have indicated that the seriousness of the condition was an influential factor that enhanced the desire to learn more about diabetes control. There was also a gender imbalance of more participants who self-reported as female than male. This suggested that estimated effects were driven by the results for females. Unfortunately, the clinical trial was not powered to detect gender effects, and given the lack of statistical significance, a separate analysis among females is unlikely to differ much unless the males have wildly different response. This was confirmed by separately analyzing females and, to improve power, by including interactions with gender in the mixed model; the interaction effect did not attain significance. There were also more people in the intervention group who were unemployed and possibly had more time away from responsibilities to attend the intervention sessions.

Participants who self-reported having a pre-diabetes diagnosis had more emotional distress compared with those living with diabetes. Future research could explore whether such differences might be related to factors such as gaining acceptance from having managed diabetes over a greater number of years or more in-depth diabetes education and greater understanding of disease control. The collection of data for time lapse since initial diagnosis of diabetes or pre-diabetes could provide additional insights. Future research could also compare outcomes for people with high levels of social support and those with lower levels while considering urban or rural geographic residence. Older-aged participants and those who were employed part time had greater perceived self-efficacy for managing diabetes than those younger, not currently employed, or working full time. The values for diabetes fatalism, social support, and perceived diabetes self-management and their dependence on age, gender, employment status, and diabetes diagnosis may be important considerations when tailoring disease risk reduction efforts for rural AAs living with diabetes and pre-diabetes. For example, intervention strategies that can reduce diabetes fatalism may improve clinical outcomes among those managing the disease at home. As an important factor of diabetes management, social support should also be addressed [37]. The interpersonal relationships contributing to high perceived levels of social support are components of social capital resources that can positively influence desirable health behavior outcomes. Intervention approaches intended to promote and strengthen existing church, family, and friendship ties can potentially build social capital in rural communities. Another important intervention consideration would be to ensure that perceptions of diabetes self-care are congruent with actual self-care behaviors. Although perceptions of diabetes self-management may seem confident, a thorough and holistic evaluation by health professionals may reveal areas necessitating further diabetes health education and intervention.

A systematic review of randomized controlled trials showed that improved HbA1C levels were associated with culturally relevant and face-to-face interventions [48]. Public health interventions that address diabetes fatalism, social support, and perceptions of diabetes self-management in addition to impacting modifiable risk factors can be imperative for improving diabetes self-care activities and glycemic control [37]. Interventions that include social support resources such as family have been associated with improved diabetes self-management and health outcomes among people with uncontrolled glycemia [49]. Social support induces significant and sustainable declines in HbA1C levels as well as improved scores for self-management, self-efficacy, and quality of life [50,51,52,53,54] as well as reduced distress associated with diabetes fatalism [51,53]. These are important principles because people who have perceptions of confidence in self-managing diabetes are more likely to have sustainable long-term effects and adherence to recommended lifestyle behaviors [55].

The strengths of this secondary analysis include the rigor of the parent study [38], the cluster randomized design, and the inclusion of a sufficient number of churches and individual participants. Additionally, the use of the linear mixed model allowed for the random possible effect of the church to be entered into the statistical model. The three-week interval of study participation limited contamination from other media and rural community resources. However, there were limitations such as the possibility that the intervention content and materials may have been shared between participating churches and the potential lack of generalizability across different rural populations. Further, the three-week duration offered limited time to impact fatalism, social support, and perceived self-management. Future studies could include more sessions to address these variables and involve biological measures such as HbA1C levels to assess sustainability. The trial was retrospectively registered (ClinicalTrials.gov Identifier: NCT04795050).

## 5. Conclusions

Health promotion and cardiovascular disease risk reduction efforts implemented in rural community settings are valuable for promoting health and preventing disease among rural populations. This study contributes information about the psychosocial factors that affect glycemic control among rural southern AAs who are historically underserved, difficult to engage in research endeavors, and have increased risk for diabetes and related disparities. The Project POWER intervention had no significant effects on diabetes fatalism, social support, or perceived diabetes self-management variables. The precise estimates of the mean levels showed that, in the population, diabetes fatalism levels were moderate, and levels of social support and perceived diabetes self-management were high. These findings may have practical implications for health professionals and community health educators in rural clinics and health departments. Future strategies for improving diabetes outcomes can facilitate diabetes health intervention development and implementation.

## Figures and Tables

**Table 1:** Sociodemographic Characteristics.

Characteristic	Control (*n* = 71)				Intervention (*n* = 75)			
	*n*	%	M	SD	*n*	%	M	SD
Age (years)			61.6	12.8			61.8	12.9
Gender								
Male	22	31.0			14	18.7		
Female	49	69.0			61	81.3		
Employment Status								
Employed full time	28	39.4			15	20.0		
Employed part time	8	11.3			6	8.0		
Unemployed	35	49.3			54	72.0		
Diabetes Diagnosis								
Diabetes	44	62.0			43	57.3		
Pre-diabetes	27	38.0			32	42.7		
Education								
Did not finish high school	17	23.9			14	18.7		
High school/Some college	40	56.3			41	54.7		
Undergrad/Grad degree	14	19.7			20	26.7		
Diagnosis: Heart Disease								
Yes	14	19.7			19	25.3		
No	57	80.3			56	74.7		
Diagnosis: Kidney Disease								
Yes	4	5.6			3	4		
No	67	94.4			72	96		
Diabetic Retinopathy								
Yes	2	3.0			14	19.0		
No	69	97.0			61	81.0		
Family History: Diabetes								
Yes	64	90.1			60	80.0		
No	7	9.9			15	20.0		
Family History: Heart Disease								
Yes	35	49.3			41	54.7		
No	36	50.7			34	48.6		
Diabetes Management								
Diet/Exercise	28	39.4			25	33.3		
Oral Medications	22	31.0			21	28.0		
Diet/Exercise/Oral Meds	19	26.8			19	25.3		
Insulin	1	1.4			1	1.3		
Diet/Exercise/Insulin	0	0			1	1.3		
Oral Medication/Insulin	0	0			4	5.3		
Diet/Exercise/								
Oral Meds/Insulin	1	1.4			4	5.3		

**Table 2:** Comparison of study outcomes for intervention and control groups.

Variable	Control Group *		Intervention Group ^+^		Intervention Effect ^±^		
	Δ_C_	95% CI	Δ_I_	95% CI	*b*	95% CI	*p*
Diab Fatalism							
Total	−1.34	(−3.96, 1.28)	−3.15	(−5.77, −0.54)	−1.82	(−5.52, 1.88)	0.33
Emot. Distress	0.59	(−1.09, 2.26)	−1.66	(−3.33, 0.01)	−2.25	(−4.61, 0.12)	0.06
Rel-SpCoping	−1.25	(−2.75, 0.24)	−0.57	(−2.06, 0.91)	0.68	(−1.43, 2.78)	0.52
Per Self-Eff	−0.74	(−1.87, 0.39)	−0.96	(−2.08, 0.16)	−0.22	(−1.81, 1.37)	0.78
Social Support							
Total	0.11	(−0.07, 0.28)	0.22	(0.05, 0.40)	0.11	(−0.13, 0.36)	0.36
Tangible	0.13	(−0.06, 0.32)	0.14	(−0.04, 0.33)	0.02	(−0.25, 0.28)	0.90
Emot/Info	0.06	(−0.13, 0.26)	0.22	(0.02, 0.42)	0.15	(−0.13, 0.43)	0.29
Affect	0.10	(−0.10, 0.30)	0.29	(0.09, 0.50)	0.19	(−0.09, 0.48)	0.19
Pos Soc	0.15	(−0.04, 0.34)	0.27	(0.08, 0.46)	0.12	(−0.15, 0.39)	0.37
Perc Diab Self-Management	0.83	(−0.64, 2.30)	1.07	(−0.39, 2.53)	0.24	(−1.83, 2.31)	0.82

* Δ_C_ is the pretest to posttest change for the control group as estimated from the LMM. ^+^ Δ_I_ is the pretest to posttest change for the intervention group as estimated from the LMM. ^±^ *b* is the estimate of the effect of the intervention, i.e., the estimate of the coefficient for the interaction between time (pretest to posttest) and study group in the LMM (also, *b* = Δ_I_ − Δ_C_).

**Table 3:** Estimates * of population means pooled across study groups.

	Baseline		Follow Up			Population Mean Adjusted for Time	
Variable	Estimate *	95% CI	Estimate *	95% CI	*p* ^±^	Estimate ^+^	95% CI
Diab Fatalism							
Total	30.61	(28.62, 32.59)	28.35	(26.34, 30.37)	0.02	29.48	(27.63, 31.33)
Emot. Distress	15.98	(14.67, 17.29)	15.44	(14.11, 16.78)	0.38	15.71	(14.49, 16.93)
Rel-Sp Coping	7.97	(7.23, 8.72)	7.06	(6.29, 7.82)	0.09	7.51	(6.96, 8.07)
Per Self-Eff	6.58	(6.02, 7.15)	5.73	(5.15, 6.32)	0.04	6.16	(5.74, 6.58)
Social Support							
Total	4.10	(3.90, 4.30)	4.27	(4.07, 4.47)	0.01	4.18	(3.99, 4.38)
Tangible	4.05	(3.79, 4.30)	4.19	(3.93, 4.44)	0.04	4.12	(3.87, 4.37)
Emot/Info	4.09	(3.89, 4.28)	4.23	(4.03, 4.42)	0.05	4.16	(3.97, 4.34)
Affect	4.30	(4.09, 4.50)	4.50	(4.29, 4.70)	0.01	4.40	(4.20, 4.59)
Pos Soc	4.10	(3.91, 4.30)	4.32	(4.12, 4.51)	0.002	4.21	(4.02, 4.40)
Perc Diab Self-Management	28.02	(27.10, 28.93)	28.97	(28.03, 29.91)	0.07	28.49	(27.68, 29.31)

* Estimates of population means from the mixed effects model including the main effect for time. ^+^ Least squares estimates for time evaluated at the midpoint of the study intervention period. ^±^ Significance of the change from baseline to end of the study intervention period pooled over study groups.
